# Inactivation and sensitization of *Pseudomonas aeruginosa* by microplasma jet array for treating otitis media

**DOI:** 10.1038/s41522-021-00219-2

**Published:** 2021-06-02

**Authors:** Peter P. Sun, Jungeun Won, Gabrielle Choo-Kang, Shouyan Li, Wenyuan Chen, Guillermo L. Monroy, Eric J. Chaney, Stephen A. Boppart, J. Gary Eden, Thanh H. Nguyen

**Affiliations:** 1 Department of Civil and Environmental Engineering, University of Illinois Urbana-Champaign, Urbana, IL USA; 2 N. Holonyak, Jr. Micro and Nanotechnology Laboratory, University of Illinois Urbana-Champaign, Urbana, IL USA; 3 Department of Electrical and Computer Engineering, University of Illinois Urbana-Champaign, Urbana, IL USA; 4 Department of Bioengineering, University of Illinois Urbana-Champaign, Urbana, IL USA; 5 Beckman Institute for Advanced Science and Technology, University of Illinois Urbana-Champaign, Urbana, IL USA; 6 Carle Illinois College of Medicine, University of Illinois Urbana-Champaign, Champaign, IL USA; 7 Carl R. Woese Institute for Genomic Biology, University of Illinois Urbana-Champaign, Urbana, IL USA

**Keywords:** Antimicrobials, Biofilms

## Abstract

Otitis media (OM), known as a middle ear infection, is the leading cause of antibiotic prescriptions for children. With wide-spread use of antibiotics in OM, resistance to antibiotics continues to decrease the efficacy of the treatment. Furthermore, as the presence of a middle ear biofilm has contributed to this reduced susceptibility to antimicrobials, effective interventions are necessary. A miniaturized 3D-printed microplasma jet array has been developed to inactivate *Pseudomonas aeruginosa*, a common bacterial strain associated with OM. The experiments demonstrate the disruption of planktonic and biofilm *P. aeruginosa* by long-lived molecular species generated by microplasma, as well as the synergy of combining microplasma treatment with antibiotic therapy. In addition, a middle ear phantom model was developed with an excised rat eardrum to investigate the antimicrobial effects of microplasma on bacteria located behind the eardrum, as in a patient-relevant setup. These results suggest the potential for microplasma as a new treatment paradigm for OM.

## Introduction

Commonly known as a middle ear infection, otitis media (OM) is a prevalent disease associated with bacterial and/or viral pathogens in the middle ear cavity. OM affects more than 80% of children in the United States^1–5^, and the financial burden on families and healthcare providers is substantial^6,7^. If the presence of a middle ear effusion (accumulated fluid in a normally aerated and empty middle ear cavity) persists for a few months or longer, subjects may experience temporary hearing loss that impacts speech and language delay in young children. Prescription of antibiotics is a common treatment for OM, but has been shown to be ineffective in >30% of cases involving acute OM^8,9^. Surgical intervention to place a small drainage tube in the eardrum, also known as the tympanic membrane, is often the next stage of treatment for chronic or recurrent infections, but the benefits and efficacy of surgery are dependent upon the severity of OM and the specifics of the middle ear condition^10,11^. As a result, OM is both the leading infectious disease requiring antibiotics and the primary cause for the administration of general anesthesia to children^12^. The trauma of OM to patients and family, combined with the financial burden on society^3,13^, underscores the necessity for new approaches to the treatment of this infection.

Several studies have determined that chronic and recurrent OM is associated with the development of a bacterial biofilm in the middle ear space^14,15^. Biofilms are aggregated bacteria within a secreted extracellular matrix which is generally adhered to a surface. Biofilms are typically responsible for a variety of chronic infections in humans, such as chronic sinusitis, intravascular infections (on stents and catheters), and pulmonary infections^16^. Previous studies have shown the presence of a middle ear biofilm in patients with various types of OM, including chronic and recurrent OM, and OM with effusion^17,18^. It has been established that the aggregated nature of a biofilm is responsible for the lower metabolic activity of these bacteria, increased antibiotic resistance with time, and seeding recurrent infections in enclosed systems such as the middle ear cavity^14,15^. Such considerations undoubtedly account for the effectiveness of surgical interventions because the microenvironment in the middle ear cavity is physically altered^19^.

Microplasma, the name given to low-temperature plasma generated in cavities having dimensions typically in the range of 50–500 µm, is known to efficiently produce reactive atomic and diatomic molecular species which interact effectively with biological molecules^20,21^. Over the past decade, the capability of a subset of these reactive species, including OH and ^1^O_2_ (singlet oxygen), and electronically excited molecules such as N_2_* (where the asterisk denotes an excited state), in deactivating and disrupting pathogens and single-species biofilms has been well established^22–25^. The versatile designs and low operating temperature of microplasma devices, combined with the ability to localize spatially the delivery of plasma-generated species, have enabled the inactivation of bacterial pathogens for several applications in therapeutics and the environmental sciences^26–30^.

A recent study, for example, demonstrated that microplasmas produced by a jet array disrupts and reduces the viability of multiple species in 10-month-old groundwater biofilms grown within a simulated, drinking water biofilm reactor^26^. Another study involving biofilms grown from water containing corrosion inhibitors, such as silicate and tin, showed that the pore network distribution of the biofilm plays an important role in the microplasma-induced removal of biofilms^27^. Furthermore, microplasma treatment accelerated wound healing in rats in vivo^28,29^ and promoted the healing response of fungal keratitis on rabbit corneas in vivo^30^. Although previous studies suggest the potential of microplasma as an infection treatment technique, less is known of its impact on OM-related pathogens, and more importantly, its efficacy and suitability for the treatment of anatomical structures associated with the middle ear, such as the eardrum.

In this paper, compact, three-dimensional (3D)-printed microplasma jet array devices have been developed as a treatment tool for OM. The viability and antibiotic susceptibility of *Pseudomonas aeruginosa*, a common bacterial strain associated with OM, were studied for different durations of microplasma treatment. An eardrum-mimicking artificial membrane was adopted for growing *P. aeruginosa* biofilms and simulating eardrum-adherent biofilms. A middle ear phantom model was constructed with a resected rat eardrum and a chamber to simulate bacteria enclosed in the closed middle ear cavity during OM for the purpose of investigating the antimicrobial effect of free radicals or other gas phase species penetrating the eardrum. The microplasma-treated rat eardrum was monitored and evaluated by a custom-built optical coherence tomography (OCT) system in combination with histology. OCT non-destructively generates depth-resolved two-dimensional (2D) cross-sectional or 3D volumetric images of tissue with near-infrared light. Having an axial resolution of 2–10 µm, OCT is shown to be a powerful tool for non-destructively assessing the thin rat eardrum and any associated biofilm. Finally, a proposed otoscope speculum integrated with a microplasma jet array is presented as a potential tool in future studies for delivering plasma treatment to the eardrum.

## Results

### Development of a microplasma jet array

**Fig. 1:**
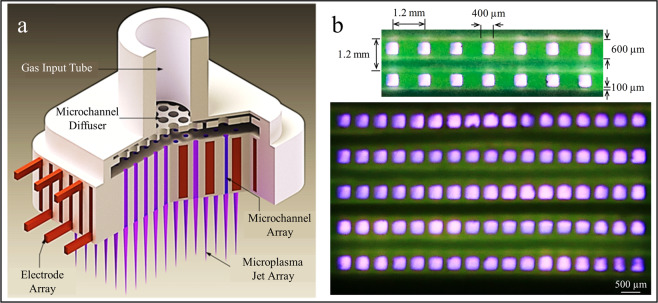
Illustration of a microplasma jet array. **a** Cutaway view of the 3D-printed microplasma jet array device and illustration of the installed electrode arrays (in red) with the array in operation, producing multiple plasma jets (in purple). **b** End-on view of two arrays of microplasma jets operating under atmospheric pressure air. Scale bar represents 500 µm.

A microplasma device capable of generating arrays of diffuse plasma jets was designed and fabricated in a transparent polymer through 3D printing. A cutaway view of a representative microplasma device structure is illustrated in Fig. 1a. Electrode arrays (shown in red) are installed into pre-assigned microchannels that extend from the bottom left corner of Fig. 1a to upper right. Microplasmas (shown in purple), generated and confined in the microchannel array, propagate from top to bottom. Figure 1b presents photographs of the microplasma array operating at atmospheric pressure with an input gas of 1% O_2_ in helium, with helium serving as the carrier gas. The top panel in Fig. 1b shows a 2 × 7 array of plasma jets emanating from channels having 400 μm × 400 μm cross-sections. The distance between neighboring channels is fixed at 1.2 mm. Microchannels of arbitrary cross-sectional geometries can readily be fabricated, and the square cross-section of Fig. 1b is presented primarily for illustrative purposes, but also to demonstrate the axial uniformity of the atmospheric-pressure glow discharges generated by the microplasma array device. For all of the studies reported here, the arrays were driven by a sinusoidal waveform with an RMS voltage of 1.55 kV, and the microplasmas dissipated a power density of 150 mW/cm^2^ over the full cross-sectional area of the array. Finally, the distance from the device to the sample was maintained at ~5 mm throughout the experiments.

### Inactivation of planktonic *P. aeruginosa*

*P. aeruginosa* inactivation was measured from the reduction of colony-forming units (CFUs) after microplasma jet array treatments were applied to planktonic *P. aeruginosa*, as illustrated in Supplementary Fig. 1a. Note that the microplasma array device was not directly applied to the colonies grown on the agar plate. After the treatment on the planktonic bacteria in a Petri dish, 100 µL of the treated bacterial solution was streaked on a brain heart infusion (BHI) agar plate and incubated at 37 °C for 16 h in order to assess the new colonies on the agar plate. After 3 min of microplasma treatment, the CFU reduction was observed to be 0.6 ± 0.3 log (~74.9% reduction). A linear reduction trend was observed for shorter exposure times, and a statistically significant CFU reduction was not observed for microplasma treatment times ranging from 3 to 7 min. However, the CFU reduction increased to 2.3 ± 0.6 log (~99.5% reduction) when the exposure time was extended to 10 min. This result is shown in Supplementary Fig. 1b. Furthermore, the diameter of the CFUs, which indicates the growth rate of the bacteria^31–33^, changed dramatically after 5, 7, and 10 min of the microplasma treatment, as indicated in Supplementary Fig. 1c.

### Antimicrobial effects of microplasma on a middle ear phantom

**Fig. 2:**
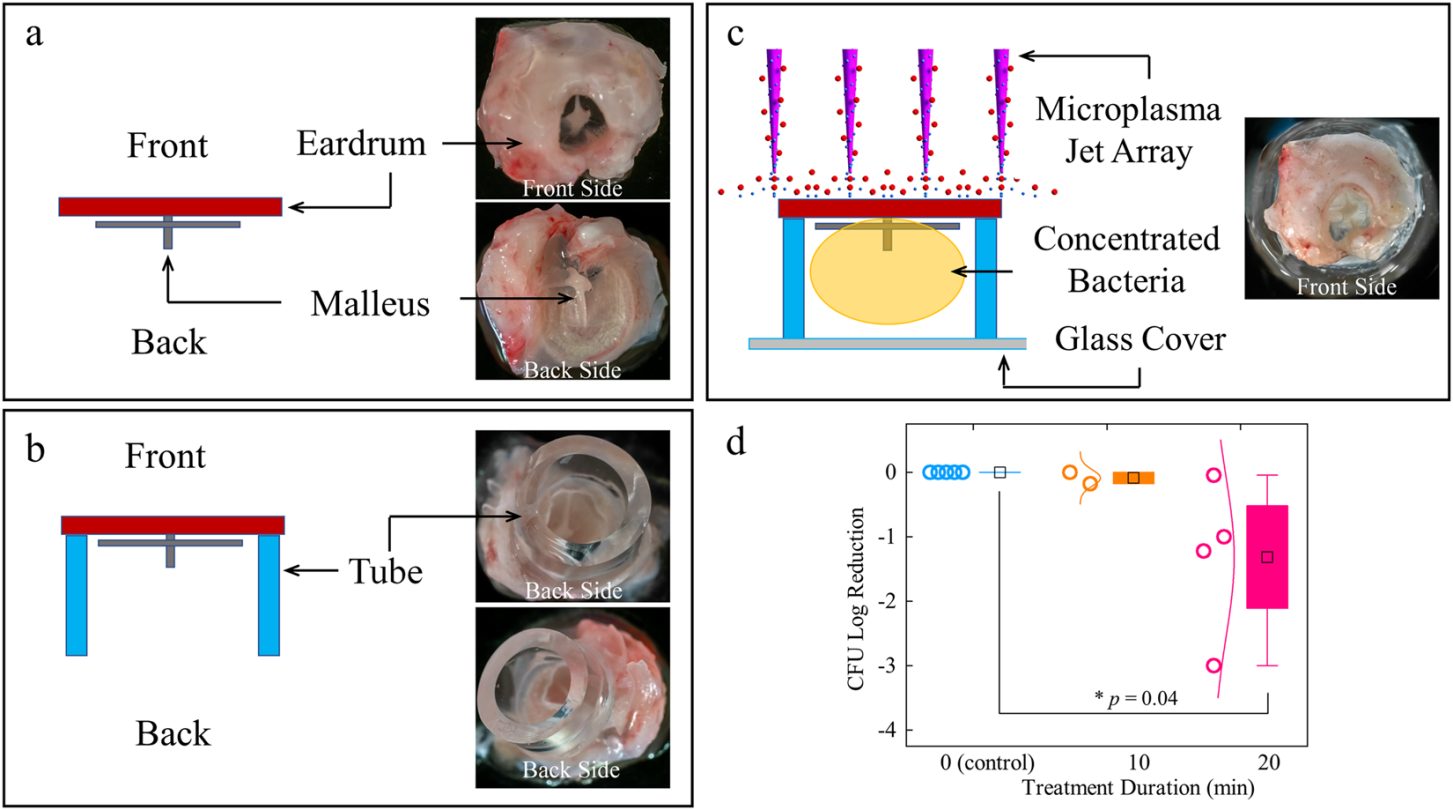
A middle ear phantom model using an extracted rat eardrum. **a** A freshly harvested rat eardrum (front and back). **b** Plastic tubing adhered to the backside of the rat eardrum to generate a middle ear cavity-mimicking space. **c** Bacterial suspension was contained in the closed middle ear cavity. **d** Plot of CFU log reduction with microplasma treatment duration.

Determining the effects of the microplasma and the generated free radicals through and behind a thin membrane, such as the eardrum, is essential if this method is to be feasible as a non-invasive, clinically viable OM treatment. Thus, a middle ear phantom model was constructed with a resected rat eardrum and chamber. Figure 2a shows a freshly harvested rat eardrum in air. In the remainder of this discussion, the frontside of the eardrum refers to the side facing the ear canal (towards the outer ear), whereas the backside of the eardrum refers to the side to which the middle ear bones are attached (i.e., towards the middle ear cavity and inner ear). Note that the overall diameter of the rat eardrum is 3–4 mm which compares to 7–8 mm for the human eardrum. Similarly, the thickness of the rat eardrum ranges from 15 to 30 µm or ~30% of that for the human eardrum (50–120 µm)^34^. To mimic the wall of the middle ear cavity, thin-walled plastic surgical tubing having an inner diameter of 3 mm was attached to the backside of the eardrum, as shown in Fig. 2b. The volume within the plastic tubing is intended to represent the middle ear cavity. A concentrated planktonic *P. aeruginosa* solution was placed inside the tubing, and the open end of the tubing was covered with a thin glass coverslip to simulate the closed middle ear cavity (Fig. 2c). This geometry is designed to determine if, and to what extent, reactive species generated by the microplasma traverse the eardrum and enter a space representative of the middle ear. The experimental procedure and microplasma operating parameters, as described above, were again adopted for this middle ear cavity phantom model. A reduction of ~90% of the CFUs was observed after 20 min of microplasma treatment (Fig. 2d), suggesting that a significant fraction of the microplasma-generated molecular radicals and excited species diffused through the eardrum and into the solution located behind the eardrum.

### Evaluation of the microplasma-treated rat eardrum by OCT and histology

**Fig. 3:**
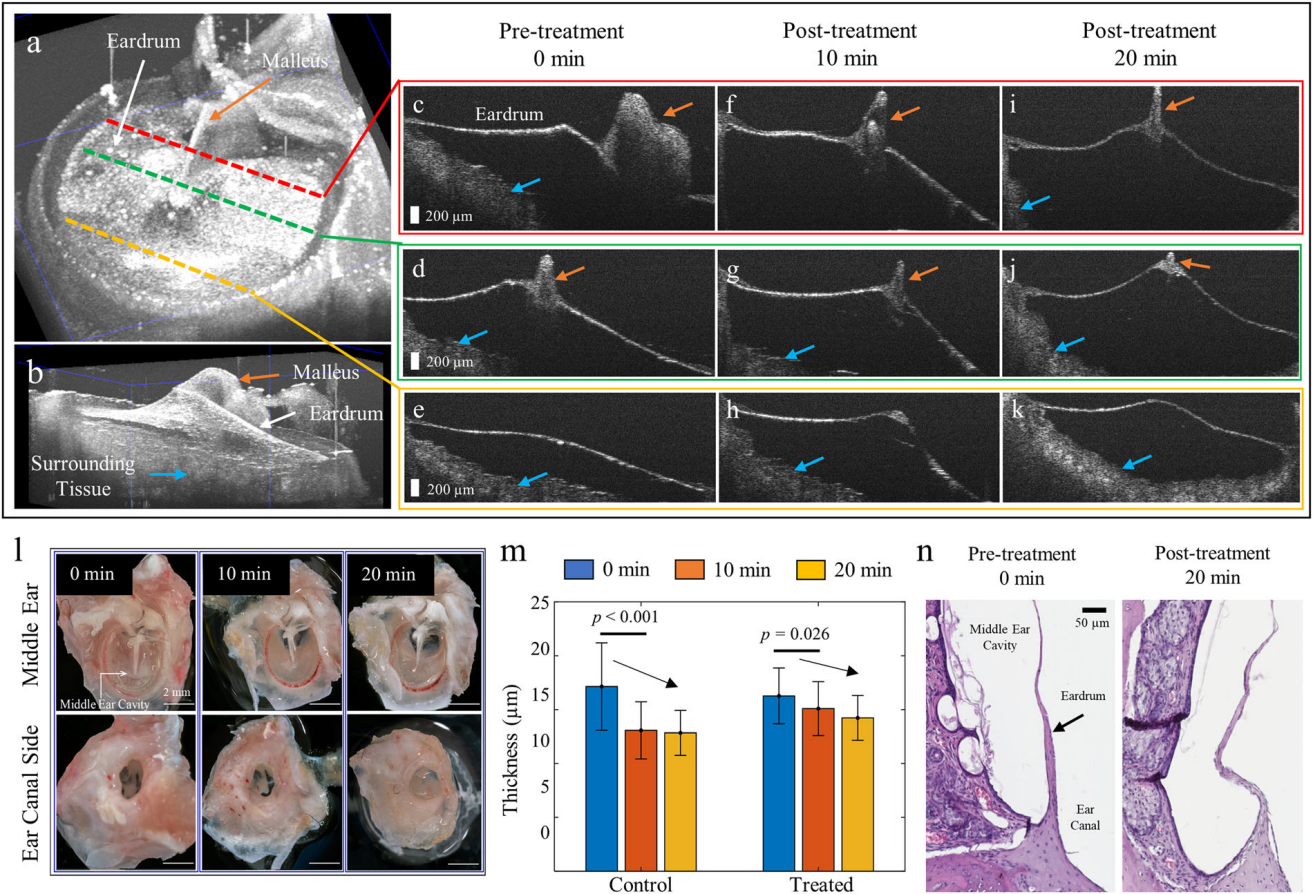
OCT and histology of the extracted rat eardrum after microplasma exposure. **a, b** A 3D reconstructed en face and cross-sectional OCT view of the excised rat eardrum. The malleus (orange arrows) attached to the eardrum is visualized. Representative cross-sectional OCT images after **c**–**e** 0 min (pre-treatment), **f**–**h** 10 min, and **i**–**k** 20 min of microplasma jet array treatments at different cross-sections. Note that the surrounding ear canal tissue (blue arrows) was captured in OCT due to the tortuous and narrow ear canal of the rat. Scale bar represents 200 µm. **l** Photos of the rat eardrum after microplasma exposure. Scale bar indicates 2 mm. **m** Plot of eardrum thickness determined from OCT with increasing duration for the rat eardrum left in air (control) and the rat eardrum exposed to microplasma (treated). The error bars denote standard deviations from different cross-sectional images. **n** Histologic images of the rat eardrum after 0 min (the pre-treatment) and 20 min of microplasma exposure. Scale bar represents 50 µm.

The rat eardrum was non-destructively examined with a custom-built, 3D volumetric OCT system after 0 (i.e., pre-treatment), 10, and 20 min of microplasma exposure. For this measurement, the middle ear phantom model described earlier was not employed since the attached plastic tubing may also affect the eardrum structures. Thus, the harvested rat eardrum, without any attachment, was directly placed into a Petri dish, and the treatment was applied to the frontside of the eardrum which faces outward as in the middle ear phantom model. Figure 3a, b presents a 3D OCT view (en face and cross-sectional, respectively) of the eardrum, recorded when the OCT beam was incident on the backside of the eardrum. Representative cross-sectional images of different regions of the rat eardrum, acquired after 0 (pre-treatment), 10, and 20 min of microplasma exposure are shown in Fig. 3c–k, respectively. Optical images of both the front and back sides of the rat eardrum are shown in Fig. 3l. No obvious rupture or physical damage to the eardrum was observed. Nonetheless, as shown in Fig. 3m, a significant decrease in the eardrum thickness was observed after 10 min of plasma exposure, as assessed by the one-way analysis of variance (ANOVA) test. It should also be mentioned, however, that a decrease in the eardrum thickness was also identified from the control eardrum left in air without any plasma exposure, suggesting that the decrease in the eardrum thickness can be attributed primarily to the dehydration of the specimen during 20 min of experiment.

Immediately after OCT imaging, the rat eardrum specimens were fixed in formalin overnight and then histologically processed for hematoxylin and eosin (H&E) staining. Representative histologic images of the pre-treated (0 min) and post-treated (20 min) specimens are shown in Fig. 3n. The pre-treatment specimen exhibited straight fibrous connective tissue within the eardrum, as compared to the treated specimen. However, several sections showed discontinuities in both pre-treated and post-treated eardrum specimens which is to be expected during histological processing for thin and delicate tissue such as that of the eardrum.

### *P. aeruginosa* biofilm on an eardrum-mimicking artificial membrane

**Fig. 4:**
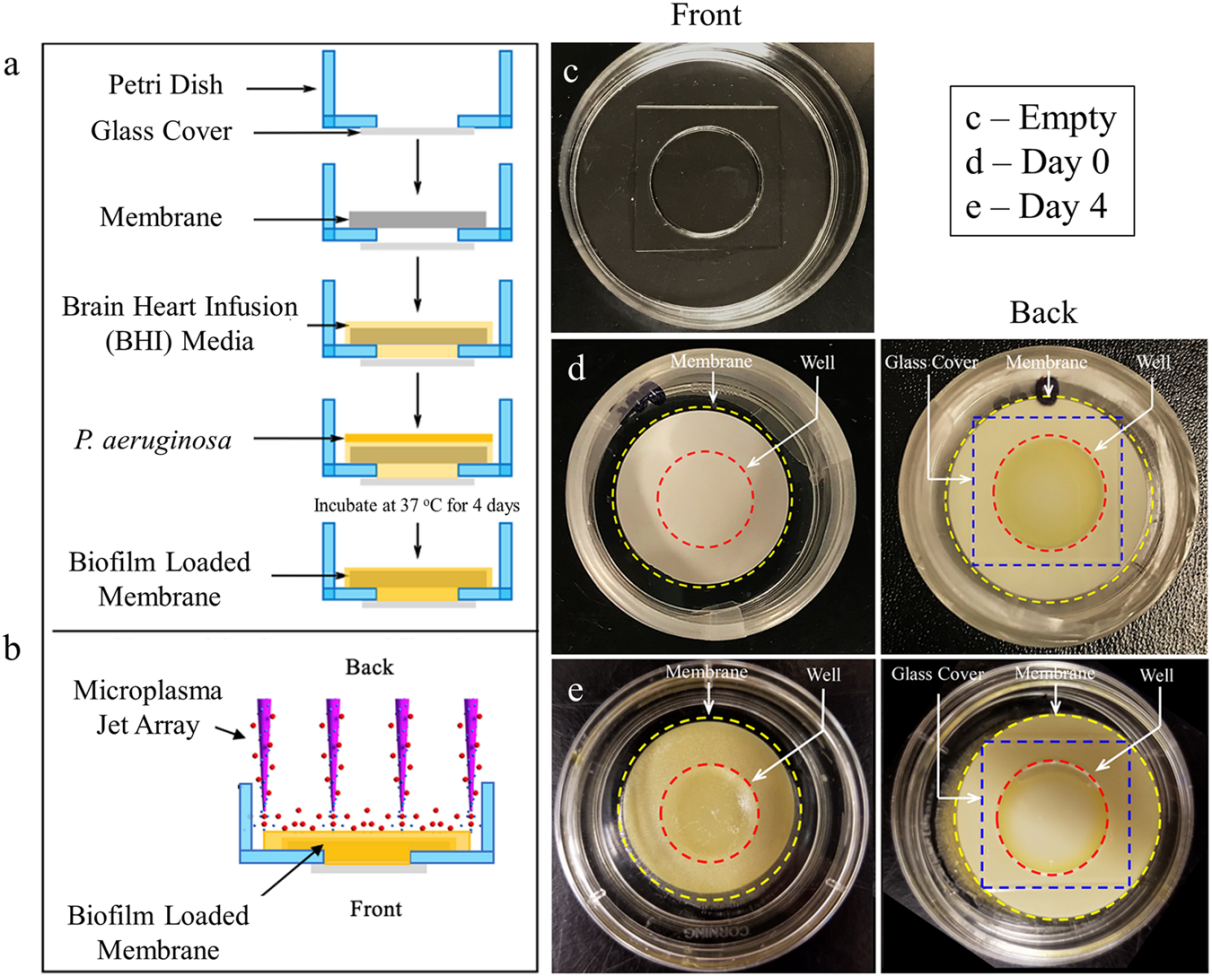
*P. aeruginosa* biofilm grown on an eardrum-mimicking membrane. **a** *P. aeruginosa* biofilm protocol for the eardrum-mimicking membrane. **b** Final diagram of the biofilm phantom and the direction of microplasma jet array treatment. **c** Photo of an empty glass bottom cell culture dish. **d** Photos of the membrane immediately after loading the bacterial suspension. **e** Photos of 4-day-old biofilm on the membrane.

In order to more closely simulate the microplasma treatment of OM in humans having a middle ear biofilm, a series of experiments was conducted with an artificial membrane onto which *P. aeruginosa* biofilms were grown. Middle ear bacterial biofilms grow not only on the inner surface of the eardrum but often exist throughout the middle ear cavity^18^. In order to mimic a thin eardrum, *P. aeruginosa* biofilms were grown on porous, biocompatible, cellulose nitrate membranes (Sartorius Stedim Biotech), shown in Fig. 4a. Because of its structure and ~130 µm thickness, these membranes are preferable to the rat eardrum as an approximation to the human membrane. Figure 4b illustrates the final biofilm phantom model designed to simulate the application of plasma on the biofilm. Figure 4c is a photograph of an empty glass bottom dish, while Fig. 4d shows the biofilm-membrane growth process immediately after loading the *P. aeruginosa* suspension. Figure 4e shows that same membrane after incubating for four days at 37 °C. Note the appearance of a yellow *P. aeruginosa* biofilm at the center of the membrane (denoted by the dashed red lines), formed as a result of trapping the bacterial suspension in the well of the plate. The edge of the membrane and coverslip at the bottom are indicated by yellow and blue dashed lines, respectively.

**Fig. 5:**
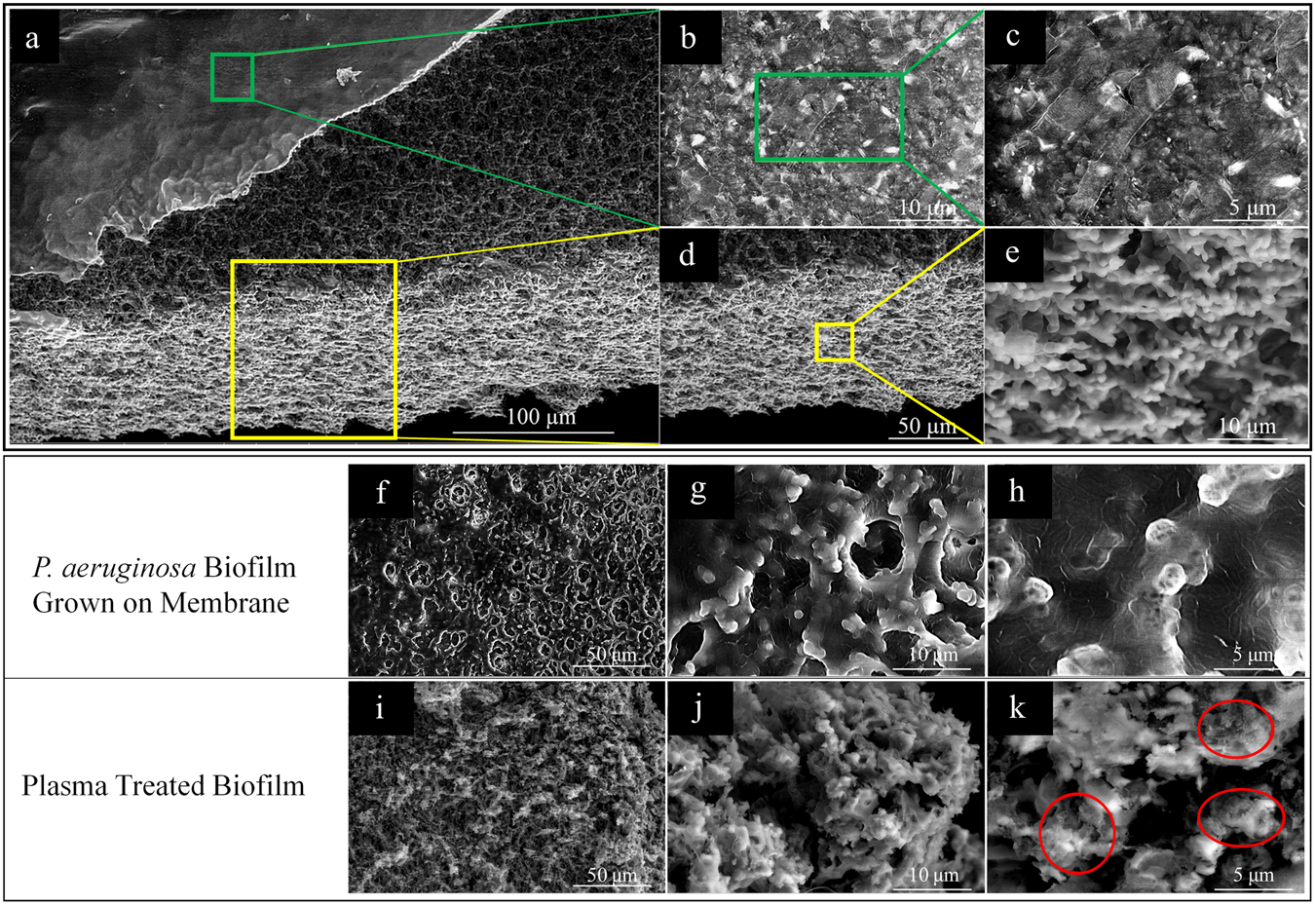
SEM of *P. aeruginosa* biofilm grown on the eardrum-mimicking membrane. **a** *P. aeruginosa* biofilm on the top side (top left corner, green box) and the cellulose nitrate membrane on the bottom side (lower center, yellow box). Scale bar represents 100 µm. **b**, **c** Magnified images of the green box in **a**. **d**, **e** Magnified images of the yellow box in **a**. **f**–**h** *P. aeruginosa* biofilm formed on the cellulose nitrate membrane. **i**–**k** *P. aeruginosa* biofilm on the membrane after 20 min of the microplasma treatment.

To validate and assess the presence and structure of the biofilm, the membrane of Fig. 4 was examined by a scanning electron microscope (SEM) and several representative micrographs are presented in Fig. 5a. Two different morphologies were observed from the top of the membrane (Fig. 5a–c, green box): the first in the vicinity of the *P. aeruginosa* suspension and the second was located at the bottom of the membrane (Fig. 5a, d, e, yellow box) where the BHI medium had solidified. The pre-solidified medium, together with the *P. aeruginosa* suspension, filled the porous network on the membrane. The larger pore size of the membrane (8 μm), as compared to that of the bacteria (~3 μm in length), allowed for *P. aeruginosa* to grow within the porous network of the membrane. No intact bacterial cells were observed after the microplasma jet array treatment, as shown in Fig. 5f–h. It is presumed that reactive species, such as OH and ^1^O_2_ produced by the microplasma jet array^26^, or electronically excited species having long lifetimes, are responsible for the disruption of the biofilm itself as well as the matrix. Because the O_2_/He plasma exiting the jet array subsequently interacts with ambient air, it is likely that additional ground and excited species, including NO and electronically excited N_2_, are also contributing to the destruction of bacteria. Collapsed structures were also observed (red circled regions) in Fig. 5i–k.

### Antibiotic susceptibility of planktonic *P. aeruginosa* and its biofilm

**Fig. 6:**
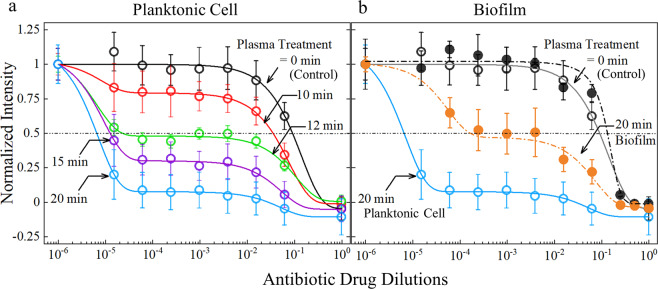
Antibiotic susceptibility measurements of *P. aeruginosa*. **a** Antibiotic drug susceptibility for microplasma-treated planktonic P. aeruginosa, with control data compared to 10, 12, 15, and 20 min of microplasma treatment. **b** Susceptibility measured for microplasma-treated four-day-old *P. aeruginosa* biofilm. The intensity is the normalized absorbance from the XTT assay. The error bars denote standard deviations from the triplicate samples.

The antibiotic susceptibility of microplasma-treated *P. aeruginosa* planktonic cells and the biofilm grown on the eardrum-mimicking membrane were measured pre- and post-exposure to microplasma. With 10 min of microplasma treatment, the 50% minimal inhibitory concentrations (MIC_50_) of the antibiotics (A5955, Sigma-Aldrich Co., St. Louis, USA) against the planktonic cells were found to decrease from 9 × 10^−2^ dilution to 3.4 × 10^−2^ dilution (i.e., a reduction of ~62%). As the treatment duration was raised to 12, 15, and 20 min, the MIC_50_ declined to 2.28 × 10^−5^ (~97.5% reduction), 1.24 × 10^−5^ (~98.6%), and 6.47 × 10^−6^ dilutions (~99.3%), respectively, as shown in Fig. 6a. These results demonstrate clearly that microplasma jet array treatment of planktonic bacterial cells reduces the required antibiotic dosage by as much as three orders of magnitude, as compared to the control group without treatment.

Similarly, Fig. 6b illustrates the antibiotic susceptibility of *P. aeruginosa* biofilms after 20 min of microplasma treatment. The biofilms grown on the eardum-mimicking membrane were washed off, followed by vortexing and pipetting in phosphate-buffered saline (PBS). The microplasma was subsequently applied to the biofilm suspension, in a manner identical to that for the planktonic bacteria (illustration in Supplementary Fig. 1a). The black dashed line represents the antibiotic susceptibility response of the control group (i.e., no treatment). Compared with the MIC_50_ value for the planktonic cells, the corresponding value for the biofilms was found to be a factor of ~1.44 larger (1.33 × 10^−1^ dilution), which is expected as the biofilm acts as a shelter for the bacteria from external conditions and pressures^35^. Nonetheless, the MIC_50_ value for the biofilm decreased by ~3 orders of magnitude (1.62 × 10^−4^ dilution) after 20 min of treatment, when compared to the biofilm control (Fig. 6b). These results suggest that microplasma treatment significantly reduces the viability of both *P. aeruginosa* planktonic cells and biofilms, thereby leading to a significant synergistic effect with antibiotics.

### Proposed microplasma jet array integrated otoscope speculum

**Fig. 7:**
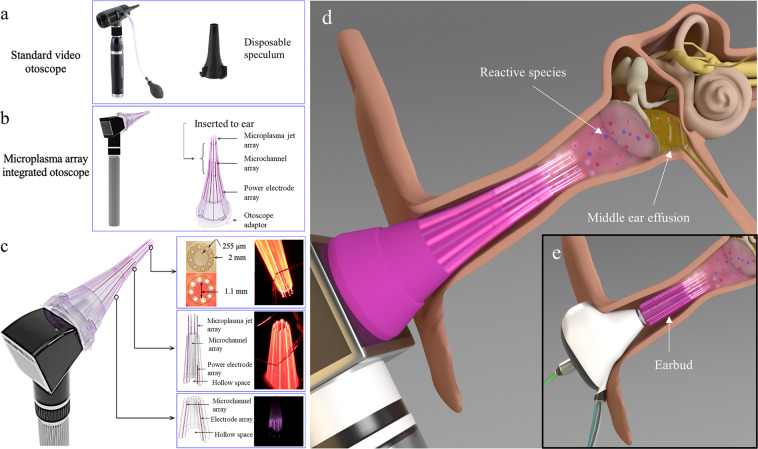
Designs of microplasma delivery platforms for the ear. **a** A standard otoscope and its disposable speculum. **b** Representative view of the otoscope speculum integrated with microplasma jet array. **c** Diagrams and photos of the speculum head with neon microplasma jets produced in the microchannels. The panels show the end-on, cross-sectional view of the speculum head, and the conical microchannel array that can alternatively be used for gas delivery and power electrodes. **d** Illustration of the microplasma-integrated otoscope delivery platform in the ear. **e** Illustration of an earbud-based microplasma delivery platform in the ear for longer treatment durations. The hollow center space for both designs in **d**, **e** allows for visual access and information.

The microplasma-based treatment reported here can be more efficiently translated to in vivo animal models and humans if the delivery platform can be integrated into a standard diagnostic tool, such as an otoscope (Fig. 7a). The preliminary concept of a microplasma-integrated otsocope speculum was based on a replaceable, 3D-printed microplasma jet array produced in the shape of an otoscope speculum, as shown in Fig. 7b. The device comprises four components: the adapter to the otoscope, the gas feed network, the electrode array, and the microplasma array itself. An end-on view of the speculum head is illustrated in Fig. 7b. Eight microchannels, each with a diameter of 255 μm, are equally distributed around the concentric speculum, as shown by the images in the right panels of Fig. 7c. The hollow speculum has an outer and inner diameter of 2 and 1.1 mm, respectively. The hollow central space allows for the transmission of light so as to allow for visual diagnosis of the eardrum by conventional otoscopy. The speculum also contains conical arrays of microchannels to accommodate electrodes and the introduction of gas through alternate channels, as shown in Fig. 7c. Different illustrations of microplasma delivery platforms are shown in Fig. 7d, e.

## Discussion

In this study, a miniaturized 3D-printed microplasma jet array was developed to investigate the disinfection and sensitization of *P. aeruginosa* with the intent of developing a potential antibiotic-free treatment for OM. While antibiotics are a common treatment regimen for OM, antibiotics are not always effective^8^. Although the free radicals and excited molecular and atomic species generated by the microplasmas have been known to deactivate various strains of bacteria^23,24^, and to do so with no known negative effects on healthy tissue^36^, its feasibility as a potential treatment paradigm for OM has not been investigated previously. In order to simulate middle ear conditions during OM, this study explored the effects of microplasma on both planktonic *P. aeruginosa* and its biofilms, adherent to thin artificial and natural membranes.

The in vitro experiment showed effective *P. aeruginosa* inactivation by measuring the reduction of CFUs and the diameters of colonies following microplasma exposures of differing durations. It should be emphasized that although a 0.6 log (74.9 %) reduction of CFUs was achieved within the first 3 min, the colony size remained the same. When the treatment time was extended to 5 and 7 min, no significant reduction of CFUs was observed, but the colony size decreased dramatically (*p* = 0.002). Both the number of CFUs and colony size dropped when the treatment duration was extended to 7 and 10 min (*p* « 0.05). These observations suggest the importance of assessing both the diameter of colonies and the reduction of CFUs to determine the degree of inactivation of bacterial strains.

Furthermore, a biocompatible and porous cellulose membrane with a thickness of 130 µm was adopted as an eardrum-mimicking phantom on which a *P. aeruginosa* biofilm was grown (Fig. 4). We validated the presence of biofilm adherent to the membrane and applied microplasma. The antibiotic susceptibility measurement showed that the MIC_50_ value for the biofilm was significantly decreased relative to that of the control. In this experiment, however, note that the microplasma was directly applied to the backside of the membrane on which the biofilm was grown. Without the pre-solidified agar medium on the cellulose membrane, the biofilm did not grow thick and adhere to the membrane. However, as the pre-solidified agar medium filled the porous network of the membrane, the inactivation from the treatment on the frontside of the membrane was not significant.

An ideal, non-invasive OM treatment will necessarily be applied from the ear canal, penetrate the eardrum, and enter the middle ear cavity. Thus, assessing the efficacy of microplasma treatment on bacteria located behind a thin membrane was crucial. Our study demonstrated a patient-relevant setup. A complete middle ear cavity phantom consisting of an excised rat eardrum and surgical tubing, designed to simulate bacteria enclosed in the middle ear cavity during OM, was developed (Fig. 2). To our knowledge, the results presented here represent the first demonstration of the antimicrobial effect of microplasma on bacteria in a simulated middle ear cavity, realized by the penetration and diffusion of free radicals and other atomic and molecular species through eardrum tissue. This conclusion is also supported by previous studies of the penetration depth of reactive oxygen and nitrogen species through hundreds of micrometers of muscle tissue^37^ and a gelatin model^38^.

As a potential treatment technology, the deactivation of biofilms by exposure to microplasma requires characterizing the impact of microplasma on healthy tissue. Although several studies have demonstrated operational parameters that minimize plasma-associated damage to healthy tissue while effectively controlling bacteria and biofilms^36,39–41^, the effect of plasma on the thin eardrum has not previously been verified. Consequently, extracted rat eardrums exposed to microplasma for several different periods of time were examined with non-destructive, structural OCT imaging, followed by histology (Fig. 3). Although there was no discontinuity (perforation) of the eardrum after 20 min of microplasma treatment, the eardrum thickness estimated from OCT images decreased with rising treatment duration. However, a decrease in the eardrum thickness was also observed from the control sample left in air without any microplasma treatment. This is likely a result of desiccation of the ex vivo tissue in air during the experiments. In addition, the thickness of the rat eardrum was observed to vary from 10 to 40 μm over its surface^42^. Thus, additional functional testing with in vivo animal models, followed by thorough histological examination, will be necessary to determine precisely the impact of microplasma treatment on the eardrum, the epithelium of the ear canal, and the middle ear mucosa.

Based on the promising inactivation effects of the microplasma on *P. aeruginosa*, we designed and prototyped a new otoscope speculum that is integrated with a microplasma jet array (Fig. 7). This device has been designed to be integrated with an otoscope, the standard diagnostic tool for the ear. Furthermore, open ports or channels are included in the otoscope design so as to allow any air or other gas to pass out of the ear canal, and prevent pressure build-up during the treatment^43^. In the future, the pressure generated from the plasma can be measured and monitored. Advanced optical imaging technologies for the eardrum and middle ear, such as OCT^44^, can be implemented through the hollow central lumen of the speculum for visualizing and characterizing the eardrum during microplasma treatment. It is expected that treatment can be controlled while simultaneously and optically monitoring the changes in the middle ear structure brought about by the microplasma^27^. However, this handheld design may pose clinical challenges with longer duration treatments, and in vitro and in vivo animal studies will be necessary to determine the optimal parameters for effective, yet safe treatment. As an alternative delivery platform for longer exposures, a microplasma array integrated into earbuds has also been designed and is illustrated in Fig. 7e. Finally, we note that the lifetimes of the molecular radicals and excited species of interest here can reach hundreds of milliseconds and beyond which should allow for the microplasma generator to be located as much as several meters from the patient. The molecular products of the plasma could then be transported to the patient’s ear with small diameter polymer tubing.

Although this study demonstrates the feasibility and potential of a microplasma jet array as a treatment method for OM from in vitro and middle ear phantom studies, several limitations of the present study must be addressed in the future. First, only one type of bacteria associated with OM was investigated. The effect of microplasma on the three other dominant strains of OM, *Haemophilus influenzae, Streptococcus pneumoniae*, and *Moraxella catarrhalis*, and their mixed cultures, should be studied. Second, the in vitro biofilm phantom and middle ear phantom models showed promising therapeutic effects of microplasma, but additional investigations with in vivo animal models for OM are also necessary. We also wish to reiterate that the rat eardrum utilized in this study is 2–4 times smaller in scale (in both diameter and thickness) than the human eardrum. Finally, the OCT and histologic evaluation of the thin eardrums was limited in the sense that additional studies to more thoroughly examine the safety profile of different microplasma intensities (i.e., molecular radical and excited specie production rates) and treatment durations are desirable. A few studies have shown that there are no side effects of plasma on skin and mucosa^45–47^, but no such studies have yet been conducted on eardrum tissue, to our knowledge. The cytotoxicity largely depends on the treatment duration, type of tissue, plasma device configuration, and plasma characteristics. Cytotoxic effects or strong interference of plasma-generated reactive species with normal cell proliferation in the eardrum and the surrounding tissue could cause potential damage to the function of the ear, such as impaired hearing. However, this remains to be investigated. Thus, in the future, acute and long-term cytotoxic effects of the current device configuration and parameters on healthy epithelial and mucosal cells from the ear will be examined in 3D cell cultures as well as in preclinical animal models.

The limitations of the present study suggest the need for reliable functional testing on in vivo animal models as well as more accurate phantom models of the ear in the future. As a next step, we will employ a 3D bio-printed eardrum model or implantable eardrum scaffolds that highly resemble the human tympanic membrane so as to better represent and examine the structures and properties, including tissue permeability. In vivo preclinical models, such as otitis media-induced chinchilla models, will also be helpful to evaluate the efficacy of microplasma on naturally grown middle ear biofilm that is adhered to the eardrum^48^, as well as on the mucosal membrane. Different designs of microplasma delivery platforms developed in the present study (Fig. 7d, e) will be beneficial to investigate the impact of microplasma in vivo. Furthermore, other factors that impact the use of microplasma on the human eardrum must be assessed, including measurements of pneumatic leakage or water permeability, and determining changes in the mechanical properties of the eardrum, such as its stiffness and mobility^49^.

Finally, we note that the treatment duration depends on the bacterial density, which is related to the disease severity and the volume of ear effusions, and dependent on the different operating parameters of plasma, such as the driving voltage, power density, frequency, input gases, and device geometries. Future studies will investigate different plasma chemistry to inactivate bacteria more efficiently in a shorter treatment duration by controlling plasma-generated reactive species.

## Methods

### Microplasma jet array device

The microplasma jet array, configured in a 9 × 9 pattern, was fabricated in a transparent polymer through 3D printing. Helium and 1% of oxygen mixed gas was used as the input gas with a flow rate of 0.25 slm/microchannel (~25 slm). The device was powered by a 1.55 kV (RMS) 20 kHz AC waveform to achieve a power density of 150 mW/cm^2^.

### Bacterial cultures

*Pseudomonas aeruginosa* (ATCC 14203) was propagated in BHI solution at 150 r.p.m. at 37 °C for 16 h, and repropagated with 1 mL into 10 mL of fresh BHI solution for 2 h at 200 r.p.m. before experiments, to ensure the exponential growth. For the experiments with planktonic bacteria, 100 μL of the *P. aeruginosa* solution in the exponential growth phase was added into 10 mL PBS. After microplasma jet array treatment, 100 μL of each treated sample was uniformly spread on a BHI agar plate and kept at 37 °C for 16 h to form the CFUs (illustration shown in Supplementary Fig. 1a). The number of CFUs was measured to evaluate the inactivation efficiency. The diameters of the CFUs were measured to evaluate the cell growth rate^31–33^. Note that the microplasma was not directly applied to the pre-grown colonies on the agar plate.

### Biofilm on eardrum-mimicking phantom

Four-day-old *P. aeruginosa* biofilm was prepared on the cellulose nitrate porous membrane (a thickness of 130 µm and a diameter of 25 mm, Sartorius Stedim Biotech) to simulate the biofilm adherent to the eardrum, as shown in Fig. 4. Both sides of the membrane were exposed to the oxygen plasma (Harrick Plasma Oxygen Cleaner) under power input of 15 W for 2 min to improve the hydrophilicity. To provide enough nutrient for the biofilm formation, 700 μL of sterilized BHI media was added to the membrane surface to form solidified media inside the membrane. Then, 200 μL of the *P. aeruginosa* solution in the exponential growth phase was added onto the surface of the membrane with the solidified media and incubated at 37 °C for 4 days. To validate the presence of biofilm and observe its structural changes due to the microplasma, the membranes were imaged with an environmental scanning electron microscope (FEI Quanta FEG 450 ESEM). The antibacterial susceptibility measurement was performed on the biofilm grown on this eardrum-biofilm model, using the XTT assay (see “Antibacterial susceptibility measurement“).

### Tissue preparation for a middle ear phantom model

All animal care and handling procedures were conducted under a protocol approved by the Institutional Animal Care and Use Committee (IACUC) at the University of Illinois at Urbana-Champaign. In this study, a total of nine Sprague Dawley rats (Envigo) at 10 weeks of age were used. Eardrums were harvested immediately after euthanasia by CO_2_ inhalation. In order to maintain intact tympanic membranes (eardrums), the tympanic membranes were isolated by dissecting the skull, instead of harvesting through the ear canal. The dissection was performed by first removing the temporal bone and the skull. Once the middle ear cavity was exposed, the eardrum along with the tissues surrounding the tympanic annulus was acquired. The specimens were stored in PBS at −4 °C until the experiments. Note that malleus bone was left attached to the tympanic membrane, but the incus bone was separated from the malleus and not included in the specimen. Specimens were used no later than 2 h after the tissue was harvested.

### Optical coherence tomography

For the non-destructive assessment of the extracted rat eardrum, a custom-developed, spectral-domain OCT was utilized to capture depth-resolved volumetric images of the specimens. A superluminescent diode with a wavelength of ~1325 ± 50 nm (Thorlabs, New Jersey) was used as a light source. The axial and lateral resolution were determined to be around 8 and 16 μm in air, respectively. A spectrometer with an InGaAs line-scan camera (Goodrich, North Carolina) enabled an A-scan rate of around 92 kHz. An imaging depth of 2.3 mm was achieved in air. A volume of 4 mm (width) by 4 mm (height) by 2.5 mm (depth) was acquired using a 2D galvanometer scanner (Thorlabs, New Jersey) with a total of 200 B-scans per volume. OCT images were processed using MATLAB and rendered using Voxx. More detailed schematics of this OCT system can be found in Huang et al.^50^. To compute the thickness of the eardrum, the OCT images were segmented to select the eardrum (excluding regions near the umbo and malleus) after median filtering and conversion to binary images with thresholding. The estimated physical thickness was computed, assuming a refractive index of 1.44 for the tissue^34^, and was statistically analyzed using a one-way analysis of variance (ANOVA) test in MATLAB. The estimated thickness less than 10 μm was excluded from the statistical analysis, as the OCT system resolution in depth was around 8 μm.

### Histology of extracted rat eardrum

After OCT imaging, rat eardrums were fixed in neutral-buffered formalin overnight and were decalcified for 4–6 h. The tissues were embedded in paraffin and sectioned at 5 μm (thickness) using a microtome (Leica Microsystems). The tissue sections were stained with H&E, which stained cell nuclei blue and stained cytoplasm and extracellular matrix pink.

### Antibacterial susceptibility measurement

An antibacterial susceptibility test was performed according to a method described previously^51,52^. For this study, antibiotics (A5955, Sigma-Aldrich Co., St. Louis, USA) consisting of 62.5 μg of penicillin, 100 μg streptomycin, and 0.25 μg amphotericin B per mL were used for susceptibility measurements. Fifty microliters of the serially diluted antibiotic solutions were added into a 96-well plate containing microplasma-treated planktonic bacteria. For biofilm, the biofilm grown on the eardrum-mimicking membrane was washed off, followed by vortexing and pipetting three times in PBS. Then, the suspension was treated with the microplasma, as in the method for planktonic bacteria described in Supplementary Fig. 1a. The untreated bacteria and biofilms were used as controls. After incubation at 37 °C for 6 h, the metabolic activities of plasma-treated biofilms were assayed by the XTT assay.

As per the manufacturer’s protocol, the XTT labeling mixture was prepared by mixing 5 mL XTT labeling reagent with 0.1 mL electron coupling reagent (XTT, Sigma-Aldrich Co., St. Louis, USA). After the incubation period (6 h at 37 °C), 50 μL of BHI medium and 50 μL of the XTT labeling mixture were added to the samples in the 96-well microplate. The plates were covered with aluminum foil and incubated at 37 °C for 2 h. Then, the absorbance at 490 nm was measured by a microplate reader (Model 680 Microplate Reader, Bio-Rad). All samples were triplicated for statistical analysis. After subtracting the absorbance of the negative control, the MIC_50_ for the biofilm was calculated. MIC_50_ is defined as the concentration of antibiotics at which a 50% decrease in absorbance was detected, compared to the positive controls.

### Reporting summary

Further information on research design is available in the Nature Research Reporting Summary linked to this article.

## Supplementary information

Supplementary Information

Reporting Summary

## Data Availability

Data and materials used in this study are available upon reasonable request to the corresponding authors and under a collaboration agreement.

## Code availability

Computer code used in this study is available upon reasonable request to the corresponding authors and under a collaboration agreement.
