# Supplementary material for: Inactivation and sensitization of Pseudomonas aeruginosa by microplasma jet array for treating otitis media
Source: NPJ Biofilms Microbiomes. 2021 Jun 2;7:48. doi: 10.1038/s41522-021-00219-2 (PMC8172902; doi:10.1038/s41522-021-00219-2)
Supplement: Supplementary file 1 — Supplementary Information [file 41522_2021_219_MOESM1_ESM.pdf]

***Supplementary Information**

Inactivation and sensitization of *Pseudomonas aeruginosa* by microplasma jet array for treating otitis media

Peter P. Sun[1,2,3†], Jungeun Won[4,5†], Gabrielle Choo-Kang[1†], Shouyan Li[3], Wenyuan Chen[3], Guillermo L. Monroy[4,5], Eric J. Chaney[5], Stephen A. Boppart[3,4,5,6*], J. Gary Eden[2,3*], and Thanh H. Nguyen[1,6,7*]

[1]Department of Civil and Environmental Engineering, University of Illinois Urbana-Champaign, Urbana, IL 61801, USA

[2]N. Holonyak, Jr. Micro and Nanotechnology Laboratory, University of Illinois Urbana-Champaign, Urbana, IL 61801, USA

[3]Department of Electrical and Computer Engineering, University of Illinois Urbana-Champaign, Urbana, IL 61801, USA

[4]Department of Bioengineering, University of Illinois Urbana-Champaign, Urbana, IL 61801, USA

[5]Beckman Institute for Advanced Science and Technology, University of Illinois Urbana-Champaign, Urbana, IL 61801, USA

[6]Carle Illinois College of Medicine, University of Illinois Urbana-Champaign, Champaign, IL, 61820, USA

[7]Carl R. Woese Institute for Genomic Biology, University of Illinois Urbana-Champaign, Urbana, IL, 61801, USA

† These authors have contributed equally to this work.

* Corresponding authors: thn@illinois.edu, jgeden@illinois.edu, boppart@illinois.edu

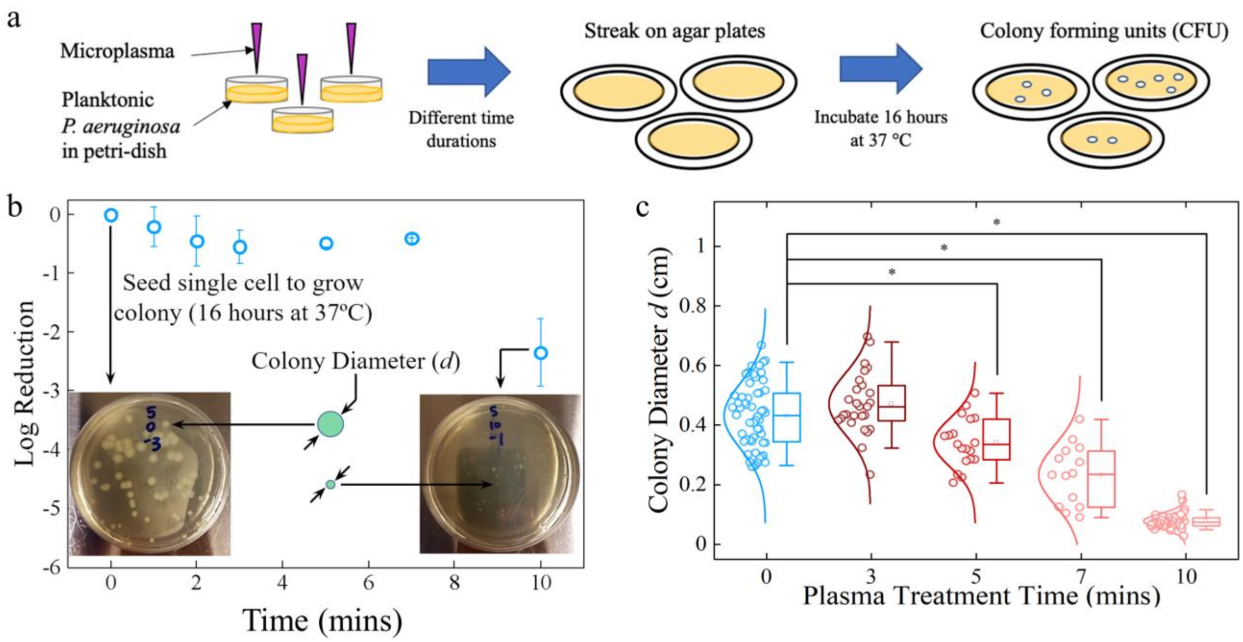

Supplementary Figure 1. Effect of the microplasma on *P. aeruginosa* colony number and diameter. **(a)** An illustration of the experimental procedure. **(b)** Plot of CFU reduction with microplasma treatment duration. **(c)** Plot of colony diameter with microplasma treatment duration. When the exposure time was increased to 5, 7, or 10 mins (with the plasma array power held constant), the colony diameter decreased to 3.4 ± 0.8 mm (p = 1.8 × 10^{-3}), 2.3 ± 0.1 mm (p << 0.05), and 0.8 ± 0.2 mm (p << 0.05), respectively.
