# Supplementary material for: Inactivation and sensitization of Pseudomonas aeruginosa by microplasma jet array for treating otitis media
Source: NPJ Biofilms Microbiomes. 2021 Jun 2;7:48. doi: 10.1038/s41522-021-00219-2 (PMC8172902; doi:10.1038/s41522-021-00219-2)
Supplement: Supplementary file 2 — Reporting Summary [file 41522_2021_219_MOESM2_ESM.pdf]

nature research

Corresponding author(s): Stephen A. Boppart
J. Gary Eden
Thanh H. Nguyen

Last updated by author(s): Mar 19, 2021

Reporting Summary

Nature Research wishes to improve the reproducibility of the work that we publish. This form provides structure for consistency and transparency in reporting. For further information on Nature Research policies, see our Editorial Policies and the Editorial Policy Checklist.

Statistics

For all statistical analyses, confirm that the following items are present in the figure legend, table legend, main text, or Methods section.

n/a	Confirmed	
☐	☒	The exact sample size (n) for each experimental group/condition, given as a discrete number and unit of measurement
☐	☒	A statement on whether measurements were taken from distinct samples or whether the same sample was measured repeatedly
☐	☒	The statistical test(s) used AND whether they are one- or two-sided<br>*Only common tests should be described solely by name; describe more complex techniques in the Methods section.*
☐	☒	A description of all covariates tested
☐	☒	A description of any assumptions or corrections, such as tests of normality and adjustment for multiple comparisons
☐	☒	A full description of the statistical parameters including central tendency (e.g. means) or other basic estimates (e.g. regression coefficient) AND variation (e.g. standard deviation) or associated estimates of uncertainty (e.g. confidence intervals)
☐	☒	For null hypothesis testing, the test statistic (e.g. F, t, r) with confidence intervals, effect sizes, degrees of freedom and P value noted<br>*Give P values as exact values whenever suitable.*
☒	☐	For Bayesian analysis, information on the choice of priors and Markov chain Monte Carlo settings
☒	☐	For hierarchical and complex designs, identification of the appropriate level for tests and full reporting of outcomes
☒	☐	Estimates of effect sizes (e.g. Cohen's d, Pearson's r), indicating how they were calculated

Our web collection on statistics for biologists contains articles on many of the points above.

Software and code

Policy information about availability of computer code

Data collection	OCT data collection was performed using custom codes in LabVIEW 2018 SP1. Other data collection did not involve computer codes.
Data analysis	Data analysis was performed using custom codes in MATLAB 2019b.

For manuscripts utilizing custom algorithms or software that are central to the research but not yet described in published literature, software must be made available to editors and reviewers. We strongly encourage code deposition in a community repository (e.g. GitHub). See the Nature Research guidelines for submitting code & software for further information.

Data

Policy information about availability of data

All manuscripts must include a data availability statement. This statement should provide the following information, where applicable:
- Accession codes, unique identifiers, or web links for publicly available datasets
- A list of figures that have associated raw data
- A description of any restrictions on data availability

Data and materials used in this study are available upon reasonable request to the corresponding authors and under a collaboration agreement.

Field-specific reporting

Please select the one below that is the best fit for your research. If you are not sure, read the appropriate sections before making your selection.

☒ Life sciences ☐ Behavioural & social sciences ☐ Ecological, evolutionary & environmental sciences

For a reference copy of the document with all sections, see nature.com/documents/nr-reporting-summary-flat.pdf

Life sciences study design

All studies must disclose on these points even when the disclosure is negative.

Sample size	Sample sizes were chosen to include at least three independent measurements. Strong inactivation caused by the plasma treatment indicated a statistical significance.
Data exclusions	When segmenting the OCT images to compute the thickness of the rat eardrum, regions near the umbo and malleus were excluded because such additional structures can increase the thickness. The estimated thickness less than 10 micrometers was excluded, as the OCT system resolution in depth was around 8 micrometers. No data were excluded for other data analysis in the study.
Replication	At least triplicate measurements of bacterial viability verified that the treatment induces decreased cell viability.
Randomization	Samples were allocated random for the study.
Blinding	Blinding was not possible since researchers directly provided the treatment and measured the efficacy.

Reporting for specific materials, systems and methods

We require information from authors about some types of materials, experimental systems and methods used in many studies. Here, indicate whether each material, system or method listed is relevant to your study. If you are not sure if a list item applies to your research, read the appropriate section before selecting a response.

Materials & experimental systems

n/a	Involved in the study	
☒	☐	Antibodies
☒	☐	Eukaryotic cell lines
☒	☐	Palaeontology and archaeology
☐	☒	Animals and other organisms
☒	☐	Human research participants
☒	☐	Clinical data
☒	☐	Dual use research of concern

Methods

n/a	Involved in the study	
☒	☐	ChIP-seq
☒	☐	Flow cytometry
☒	☐	MRI-based neuroimaging

Animals and other organisms

Policy information about studies involving animals; ARRIVE guidelines recommended for reporting animal research

Laboratory animals	Sprague Dawley female rats (Envigo) at 10 weeks of age were used.
Wild animals	The study did not involve wild animals.
Field-collected samples	The study did not involve field-collected samples.
Ethics oversight	A protocol was approved by the Institutional Animal Care and Use Committee (IACUC) at the University of Illinois at Urbana-Champaign.

Note that full information on the approval of the study protocol must also be provided in the manuscript.
